# Changes in P1 latencies of children with normal hearing and those with cochlear implants

**DOI:** 10.3906/sag-1910-233

**Published:** 2020-06-23

**Authors:** Emre ESKİCİOĞLU, Günay KIRKIM, Selhan GÜRKAN, Serpil MUNGAN DURANKAYA, Tahsin Oğuz BAŞOKÇU, Enis Alpin GÜNERİ

**Affiliations:** 1 Unit of Hearing, Speech and Balance, Department of Otorhinolaryngology, School of Medicine, Dokuz Eylül University, İzmir Turkey; 2 Department of Assessment and Evaluation in Education, School of Medicine, Ege University, İzmir Turkey; 3 Department of Otorhinolaryngology, School of Medicine, Dokuz Eylül University, İzmir Turkey

**Keywords:** Cortical auditory evoked potentials, cochlear implantation, P1 latency, children, normal hearing

## Abstract

**Background/aim:**

The aim of this study was to determine the age-related latency interval of P1 latencies of children with normal hearing, and to evaluate the P1 latency changes after surgery in children who underwent cochlear implantation.

**Materials and methods:**

We evaluated 60 children with normal hearing and 16 children with cochlear implants aged 0–6 years using cortical auditory evoked potentials. P1 latencies were measured only once in the children with normal hearing, and on the postoperative first day, and the first, third, and sixth postoperative months in the children with cochlear implants.

**Results:**

There was a statistically significant decrease in the P1 latencies as the age increased in children with normal hearing (P < 0.001). It was determined that when the external partof the cochlear implant was applied, the P1 latencies of children with cochlear implants were significantly longer than those of age-matched children with normal hearing (P < 0.001). This difference disappeared in 10 children with implants at the third and sixth months, but significant differences remained in 6 children.

**Conclusion:**

P1 latency could be used as an objective tool to evaluate the normal development of auditory pathways, and may be helpful in the effective programming of children undergoing cochlear implantation.

## 1. Introduction

Cortical auditory evoked potentials (CAEP) are thought to reflect the activities of excitatory postsynaptic potentials at the level of the thalamus and auditory cortex [1]. Although CAEP includes P1, N1, and P2 components, N1 and P2 are not considered to be reliable until 7 years of age [2]. The latency of P1 was reported to be measured within 300 milliseconds (ms) in newborns and infants, with a rapid decrease down to 200 ms at 2 years of age and maintained at 100 ms in adulthood [3–5]. This change represents the increased speed of synaptic propagation of the central auditory pathways [6].

Structural and functional changes in the brain caused by the lack of auditory stimuli can be reversed in childhood because it is possible to stimulate the auditory pathways through cochlear implants (CI) [7–10]. Phonetic perceptions of babies who have been exposedto voice stimuli during the first 6 months of their lives have been shown to produce a positive effect on the development of the auditory system [11]. Most children who undergo early implantation have normal P1 latencies relative to their age, and the P1 latencies of children who underwent implantation after 7 years of age were found to be approximately 100 ms delayed [9].

CAEP measurements are used to define CI candidates, optimise the processes, and in the follow-up of CI users [12]. In children, P1 components are typically dominant in the waveform compared with adults [5,13]. Some studies demonstrated that P1 was useful to evaluate the maturation of the central hearing system [3,14]. The shortening of P1 latency with time was explained by the maturation of the hearing system, which was also related to the duration of sound exposure [15,16]. It was demonstrated that CI users had a longer P1 latency compared to children with normal hearing and this difference was eliminated by using CIs for longer periods [17].

P1 latency can be used to evaluate the evolution of the primary cortex. Many studies conducted in different age groups showed that P1 latency shortened as age increased [9,15,18–20]. Previous studies also underlined the critical influence of implantation age on P1 latency in terms of cortical maturation [16].

Various studies evaluated the latency and amplitude of P1 in children with normal hearing aged under 12 years [21,22]. The presence of P1 and its normative data can be calculated using CAEP measurements, but previous studies are particularly scarce in paediatric patients [5]. For this reason, this study aimed to acquire normative data of P1in children aged 0–6 years, and age-related changes of P1 latencies and P1 latencies of CI users.

## 2. Materials and methods

### 2.1. Participants

This study was conducted in Dokuz Eylül University Hospital Department of Otorhinolaryngology. The demographics of the children with normal hearing and CI users are given in Tables 1a and 1b, respectively. A total of 80 children were included in the study. Sixty (24 females, 36 males) children with normal hearing were identified as the control group with newborn and routine hearing tests. The controls had no prenatal, natal, or postnatal risk factors, syndromes, and/or craniofacial anomalies. CI users who fulfilled routine follow-ups were also recruited. The CI users had no auditory nerve anomaly and central pathology. The CAEPs of 20 CI users (11 females, 9 males) who underwent CI surgery were measured, as well as the control group. The exclusion criteria were lack of consent of the family, giving up using the CI, and not collaborating with routine follow-ups. All CI users used bilateral hearing aids before surgery and received language and speech therapy following surgery. One child in the normal group was excluded from the study because an artefact was observed due to movement during the test. Four CI userswere excluded from the study due to not collaborating with follow-ups. The Local Ethical Committee of the university approved the study and participants signed the informed consent form before participation.

**Table 1a:** Mean age and sex distribution of subgroups of normal hearing children.

Subgroups (months)	Mean age (months)	Sex distribution (male)	Total children
0–6	4.4 ± 1.51	0	5
7–12	7.8 ± 0.84	4	5
13–18	14.8 ± 0.84	3	5
19–24	21.0 ± 2.00	3	4
25–30	26.4 ± 0.89	4	5
31–36	33.6 ± 1.52	5	5
37–42	38.4 ± 0.55	4	5
43–48	44.4 ± 2.71	3	5
49–54	51.0 ± 1.79	4	5
55–60	57.0 ± 1.79	2	5
61–66	64.2 ± 1.91	3	5
67–72	68.4 ± 1.67	1	5

**Table 1b:** Sex and duration of hearing aid use of cochlear implanted children and the age of cochlear implant surgery.

Case	Sex	Duration of hearing aid use (months)	Age of cochlear implant surgery (months)
1	F	6	14
2	M	6	36
3	F	7	47
4	F	3	15
5	M	3	27
6	F	23	29
7	F	5	53
8	F	38	44
9	M	25	31
10	M	54	66
11	M	5	13
12	M	39	51
13	M	6	36
14	F	12	31
15	F	16	28
16	M	8	17

### 2.2. Test scheme

The CAEP measurements were recorded while the children were seated in a comfortable armchair in an acoustically isolated room. The implant fittings were updated regularly, and no noise suppression system was used. A HearLab (Frye Electronics, Inc., Beaverton, OR, USA) CAEP device was used for CAEP measurements and analysis. A HearLab calibration microphone was used for the pretest stimuli calibration. The contralateral hearing devices of the CI users were removed during the test. The children watched a muted cartoon during CAEP measurements. 

### 2.3. Stimulus characteristics

As the CAEP stimuli, low-pitched speech stimuli (/m/ 200–500 Hz), medium-pitched speech stimuli (/g/ 800–1600 Hz), and high-pitched speech stimuli (/t/ 2000–8000 Hz) were used. The stimulus time was 30 ms for /m/ and /t/, and 20 ms for /g/. The inter stimulus interval was 1125 ms.

### 2.4. CAEP measurements

Stimuli were administered under the same conditions and parameters for CI users and children with normal hearing. The stimulus was presented first at 65 dB SPL, zero-degree azimuth (nearly normal speaking sound intensity). If P1 was acquired, the stimulus intensity was decreased to 55 dB SPL; if no P1was acquired, the stimulus intensity was increased to 75 dB SPL and the test was stopped. The accepted sweep count was 200. The active electrode was located to the vertex (Cz), the reference electrode was located to the mastoid (M1 or M2), the ground electrode was located to the forehead (Fpz). The impedance of the electrodes was below 10k ohm and the test duration including all steps was a maximum of 45 min.

### 2.5. Study design

Children with normal hearing aged 0–6 years were analysed in 12 subgroups that were divided into 6-month age intervals (Table 1a). CI users were submitted to CAEP 1 day after the application of the external part of the CI, and in the first, third, and sixth months. The P1 latencies of the CI users and age-matched control group P1 latencies were compared.

### 2.6. Statistics

The independent variable used for the investigation was the age interval and the dependent variable was P1 latency. Pearson’s correlation test was used to determine the relationship between age and P1 latency. Repeated measures ANOVA was applied to determine whether a significant difference existed between P1 latency values obtained at /m/ 65, /t/ 65, and /g/ 65 dB SPL. Regression analysis was performed between the P1 latency data at 65 dB SPL. Also, age and regression curves were obtained. A cubic prediction method was used in data analysis becauseof the nature of the gathered data. The existence of a significant difference between the P1 latencies of children with normal hearing and CI users was investigated using the Mann–Whitney U test in an independent group. Also, 1-sample t-test was performed to determine the difference between normal children mean and CI children per month. The relation was assumed very weak if the R value was 0.00–0.25, weak if 0.26–0.49, medium if 0.50–0.69, high if 0.70–0.89, and very high if 0.90–1.00. All analyses were performed in SPSS version 23.0 (IBM Corp., Armonk, NY, USA).

## 3. Results

In the normal hearing group, when the P1 latency values of /m/, /t/, and /g/ at 75, 65, and 55 dB SPL were compared with regard to age, a statistically significant difference was observed (P < 0.001). The change in P1 latency values of speech stimuli and stimulus intensity in children with normal hearing is shown in the Figure. The findings revealed that as the age increased, P1 latency shortened.

The Pearson’s correlation test was conducted to determine the relation between age and /m/, /t/, /g/. A significant and negative relationship was observed between age and latency in children with normal hearing (R > 0.69; P < 0.01). The correlation coefficient and significance levels obtained at every intensity level of /m/, /t/, and /g/ with age are presented in Table 2.

**Figure:**
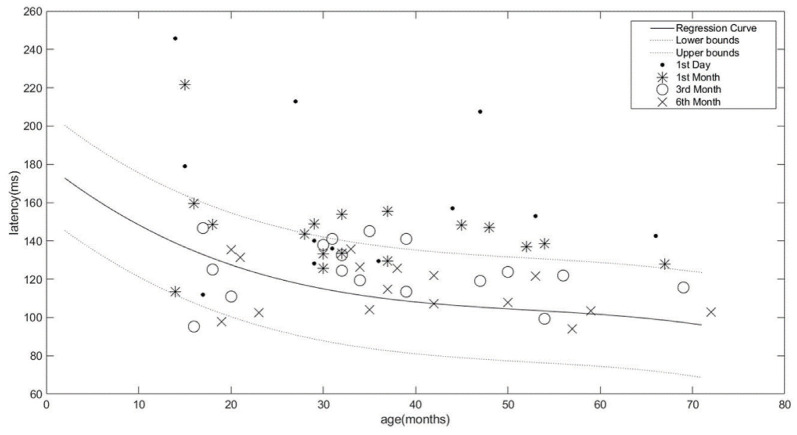
Regression curve of age and P1 latency values in normalhearing children. P1 latency changes in the rehabilitation process of cochlear implanted children. The x-axis denotes the age (months) while the y-axis refers to latency (ms).

**Table 2:** Correlation coefficients between 3 different sound intensity and speech stimuli.

Sound intensity Speech stimuli	75 dB SPL	65 dB SPL	55 dB SPL
/m/	P < 0.001 R = –0.699	P < 0.001 R = –0.773	P < 0.001 R = –0.728
/t/	P < 0.001 R = –0.716	P < 0.001 R =–0.789	P < 0.001 R = –0.765
/g/	P < 0.001 R = –0.697	P < 0.001 R = –0.756	P < 0.001 R = –0.758

A repeated measure ANOVA was conducted to assess whether there were differences between the average /m/, /t/, and /g/ P1 latency values at 65 dB SPL. Results indicated that there is no significant difference between P1 latency values, F (2,114) = 3.79, P > 0.05. 

Relying on the establishment of a nonsignificant relationship between the /m/, /t/, /g/ 65 dB SPL intensities and P1 latency values, the relationship between all speech stimuli at 65 dB SPL intensity and age were investigated together.

A significant and strong negative relationship was observed between the age and latency values when the relation between age and P1 latency values at 65 dB SPLwas investigated using Pearson’s correlation test (R > 0.769; P < 0.001). The P1 latency significantly shortened as the age increased.

Regression analysis was conducted to determine the curves of age and P1 latency values at 65 dB SPL. Results were statistically significant F (1,174) = 475.95, P < 0.01, R2, 71. The equation was created as Y = 208.56 ± 26.82*log(x) according to the regression analysis. The cubic and logarithmic model regression curves were obtained from age and P1 latency values. A cubic prediction method was used while defining the confidence interval because the data were not linear (Figure).

Both in children with normal hearing and CI users, sex had no significant impact on P1 latency obtained at /m/, /t/, /g/, and stimulus intensity using Mann–Whitney U test (P > 0.05). The P1 latency averages of CI users observed for 6 months, their age-matched peers, and the statistical analysis results are given in Table 3. A graph of the distribution of latency of the CI users on the external part application day, and in the first, third, and sixth months is presented in the Figure. It was observed that the P1 latencies of 10 CI users were in normal distribution in the sixth month for all speech stimuli. Six CI users who fell out of the normal distribution were found to have undergone CI surgery earlier than others (P < 0.001).

**Table 3:** One sample t-test results of cochlear implanted children. The mean difference was calculated by subtracting the P1 latency values of normal hearing children from the P1 latency values of age-matched peers CI children.

		Cases															
		1	2	3	4	5	6	7	8	9	10	11	12	13	14	15	16
External part application day	Normal hearing group average (ms)	137.93	144.66	107.86	137.93	120.46	120.46	100.80	107.86	114.66	100.92						124.50
	Mean difference	–107.73	–14.66	–99.63	–41.06	–92.20	–19.53	–52.20	–49.13	–21.33	–41.73						–24.50
	t	–20.29	–4.69	–61.24	–7.73	–27.33	–5.78	–19.00	–30.20	–6.83	–12.14						–5.74
	P	0.000	0.000	0.000	0.000	0.000	0.000	0.000	0.000	0.000	0.000						0.000
1st month	Normal hearing group average (ms)	137.93	104.53	107.87	137.93	120.46	120.46	100.80	107.86	114.66	96.73	137.93	100.80	104.53	114.66	120.46	124.50
	Mean difference	–83.73	–24.79	–39.13	–21.56	–23.20	–12.86	–37.86	–40.46	–19.00	–31.26	24.60	–36.20	–50.79	–39.33	–28.53	–13.50
	t	–15.72	–7.24	–24.05	–4.06	–6.87	–3.81	–13.78	–24.87	–6.08	–9.85	4.63	–13.17	–14.83	–12.60	–8.45	3.20
	P	0.000	0.000	0.000	0.001	0.000	0.002	0.000	0.000	0.000	0.000	0.000	0.000	0.000	0.000	0.000	0.008
3rd month	Normal hearing group average (ms)	137.93	114.66	100.80	124.50	120.46	114.66	103.33	107.86	114.66	96.73	137.93	100.80	104.53	114.66	114.66	124.50
	Mean difference	–8.73	1.33	–22.86	–0.50	–17.53	–9.66	–18.66	–11.13	–4.66	–18.93	42.60	1.46	–36.46	–30.33	–36.46	3.66
	t	–1.64	0.42	–8.32	–0.119	–5.19	–3.09	–10.64	–6.84	–1.49	–5.96	8.02	0.53	–10.65	–9.72	–10.65	1.17
	P	0.112	0.676	0.000	0.908	0.000	0.008	0.000	0.000	0.157	0.000	0.000	0.602	0.000	0.000	0.000	0.260
6th month	Normal hearing group average(ms)	137.93	104.53	100.80	124.50	114.66	114.66	103.33	107.86	114.66	96.73	104.53	100.80	104.53	104.53	114.66	114.66
	Mean difference	2.60	–2.79	–20,86	–6.83	–21.00	3.86	0.00	0.20	0.00	–5.93	6.86	6.92	–17.46	–21.13	–11.66	12.33
	t	0.49	–0.81	–7.59	–1.62	–6.72	2.377	0.00	–0.127	0.000	–1.87	2.00	2.01	–5.10	–6.17	–3.73	1.87
	P	0.632	0.427	0.000	0.133	0.000	0.032	1.000	0.901	1.000	0.083	0.065	0.065	0.000	0.000	0.002	0.083

## 4. Discussion

In this study, /m/, /t/, and /g/ were used as the speech stimuli in different frequencies and it was found that the impact of these speech stimuli did not differ significantly in generating the P1 latency. A few studies showed that different stimulus types, such as pure tone, click, a speech stimulus used in generating P1 did not change the P1 latency [18,22–25]. In this study, the P1 latency values were combined so that it was possible to analyse more data at the same time.

The shortening in the P1 latencies in children aged 0–6 years were divided into 6-month age intervals forming 12 subgroups in our study. The results showed a statistically significant decrease in P1 latencies as the age increased (P < 0.001). Sharma et al. [19] reported a similar trend previously. In another study conducted with 86 children with normal hearing aged 6–15 years, P1 latency was found to be negatively related to age [26]. However, Wunderlich et al. [24] reported that P1 latency shortened as age increased in children with normal hearing, although this shortening was not significant until 6 years of age. Sharma et al. [9] studied 136 children with normal hearing aged between 0.1 and 20 years of age, and they revealed a rapid shortening of P1 latency in the first 10 years of their lives; however, this shortening velocity decreased in the second decade. Dorman et al. [3] showed that this shortening during the first 3 years of life was 125 ms, whereas as lower shortening occurred in the second decade of life.

Cortical system plasticity decreases with hearing loss that persists after the age of 7 years [9,27]. In the present study, the CI age of all children was below 7 years. For this reason, most CI users were observed within normal distribution after 6 months of CI use.

In 11 CI users who underwent implantation before the age of 3 years, their shortening rate of P1 latencies was higher than those who were implanted after age 3 years. Their P1 latency reached normal distribution in 6 months. Five CI users reached the P1 latency level of normal hearing children in 3 months after the implantation. This result can be explained by the diagnosis in the first 6 months of their lives and adequate amplification with hearing aids, implantation before age 18 months, and a longer period of exposure to auditory stimuli compared with other CI users. In a study that supported the existence of a sensitive period of the auditory system, 22 children who were CI users before the age of 3.5 years were examined. After CI use for 8 months, the P1 latency of the CI users was found in the normal hearing distribution [6]. In another study, the CAEP responses of 18 children who had begun to use CI at the age of 3.5 years were compared with children with normal hearing at the same chronologic age; after 6 months’ use of CI, P1 latencies were found to be in the age group range [16]. In both studies, it was shown that the difficulties caused by a lack of hearing could be overcome with a minimally degenerated central auditory system or a central auditory system with high plasticity. 

The P1 latencies of 6 CI users at the sixth month CAEP tests were found shorter than the P1 latencies of children with normal hearing. In the study conducted by Ponton et al. [15] on CI users, the average of P1 latencies of CI users was found to be shorter than the P1 latencies of children with normal hearing. Also, this situation can be explained by the auditory stimulus having at least 2.5–3.0 ms shorter distance by passing the external ear, middle ear, cochlea, and continuing to the auditory pathways. Moreover, by direct electrical impulses provided by the cochlear implant, more nerve synchronization was enabled, and shorter latency values were reported to be obtained [15].

In this study, the P1 latencies obtained in the CAEP tests sixth months after the implantation of 6 CI users were found to be longer than the P1 latencies of children with normal hearing. Three of them were implanted late (47, 36, and 31 months), 1 used hearing aid late (23 months), and 2 did not attend auditory rehabilitation regularly. The interest of the family in the CI user, the degree of exposure to an auditory stimulus, and the duration of auditory deprivation were thought to have an impact on P1 latency values. The P1 latency of children who were diagnosed as having hearing loss before age 6 months, who were initiated rehabilitation with a hearing aid and received a CI as soon as possible, reached the levels of children with normal hearing in less than 6 months. These data support the need for early diagnosis and enabling necessary amplification without delay, as well as the need for applying CI as soon as possible.

## Acknowledgment

This study was supported by Dokuz Eylül University (DEU) Projects 2013.KB.SAG.070.
